# Acute effects of real and imagined endurance exercise on sustained attention performance

**DOI:** 10.3389/fpsyg.2022.905772

**Published:** 2022-08-30

**Authors:** Björn Wieland, Marie-Therese Fleddermann, Karen Zentgraf

**Affiliations:** Department of Movement Science and Training in Sports, Institute of Sport Sciences, Goethe University Frankfurt, Frankfurt, Germany

**Keywords:** hypofrontality, cognition, motor imagery, running, sustained attention

## Abstract

This study investigated acute effects of real and imagined endurance exercise on sustained attention performance in healthy young adults in order to shed light on the action mechanisms underlying changes in cognitive functioning. The neural similarities between both imagined and physically performed movements reveal that imagery induces transient hypofrontality, whereas real exercise reflects both transient hypofrontality effects and the global release of signaling factors (e.g., BDNF or serotonin) due to muscle contraction and the accompanying sensory feedback. We hypothesized improved cognitive functioning after both interventions (imagery and physical endurance exercise) with greater improvements for real exercise because it targets both mechanisms. Fifty-three sport science students completed two 25-min sessions of moderate endurance exercise in either a motor imagery modality or an executed bodily activity within the framework of an order-balanced crossover study. Assessments for sustained attention performance (d2-R) were performed before and after each endurance exercise condition. Statistical results showed improvements for both groups over time, which can mostly be explained by retest effects. However, we observed a significant interaction effect between group and time, *F*(1.6, 81.9) = 3.64, *p* = 0.04, *η*^2^ = 0.07, with higher increases in the first session in case physical endurance exercise was performed compared to motor imagery exercise, *t*(51) = −2.71, *p* = 0.09, *d* = 0.75. This might suggest that the release of signaling factors due to muscle contractions with sensory feedback processing is an additional mediating mechanism alongside motor-related transient hypofrontality that improves cognitive performance.

## Introduction

Being active in sports might enhance not only physical health and physical performance but also mental health (for overviews, see Biddle and Asare, 2011; Kramer, 2020). Many studies have shown that regular sport exercise can have numerous positive (mental) effects such as reducing the risk of some neurological diseases (Kramer et al., 2006), delaying the onset of age-related decline (Vivar, 2015), positively changing mood (for an overview, see Liao et al., 2015), and also improving some cognitive functions (Labban and Etnier, 2011; Prakash et al., 2015). However, it is not just regular (chronic) sport exercise that enhances mental health or performance. Even a single bout of (acute) sport exercise can also affect some mental aspects. In one of the first reviews, Tomporowski and Ellis (1986) showed that a single session of physical exercise influences cognition (e.g., cognitive tasks measuring working memory or attention). Most of the growing number of empirical studies in this field have shown short-term improvements in cognitive performance after acute exercise in, e.g., single or choice reaction tasks (López-García et al., 2019), different attention tasks (Park and Etnier, 2019), or working memory tasks (Labban and Etnier, 2011). These improvements are found especially in cognitive functions associated with prefrontal regions of the brain (Basso et al., 2015) such as those assessed with attention tasks. Moreover, there are also some reviews (Basso and Suzuki, 2017) and meta-analyses (Etnier et al., 2006; Chang et al., 2012) showing that one single exercise session has a small overall positive effect on cognitive functions. However, although there are many studies showing cognitive improvements after a single bout of exercise, comparisons between these studies and the related neurophysiological and neurochemical changes are constrained by a lack of clear and common standards (Basso and Suzuki, 2017).

There is also a growing literature examining neurochemical changes and neurophysiological changes as a result of acute exercise. Neurochemical components such as neurotrophic factors [e.g., brain-derived neurotrophic factor (BDNF) or insulin-like growth factor 1 (IGF-1)], metabolites (e.g., lactate) as well as neurotransmitters (e.g., serotonin) play an important role in brain plasticity, neuronal development, and cognitive function. Zimmer et al. (2016) studied the effects of acute exercise on serotonin and found increased serotonin levels after moderate aerobic exercise along with improved performance in a Stroop task. In addition, there is also evidence for increased BDNF levels after acute exercise in the form of moderate effect sizes (for an overview, see Szuhany et al., 2015) that correlate with enhanced cognitive performance (Winter et al., 2007).

A complementary approach to explaining cognitive improvements after exercise is based on the reticular-activating hypofrontality theory of exercise (Dietrich, 2006). Findings from a meta-analysis by Jung et al. (2021) corroborate this conceptual idea by presenting data that prefrontal-cortex-dependent cognition *during* exercise is impaired when a limited supply of energy and resources of the brain are shifted away from the prefrontal cortex to more posterior areas for movement execution. Immediately *after* the exercise session and the exercise-induced drain, there is a backward shift of oxygenated blood in the anterior regions of the prefrontal cortex. Due to this backward shift, cognitive performance could be improved. This is an explanation as to why especially cognitive functions involving the prefrontal cortex, such as attention, are often improved after acute exercise (Chang et al., 2012), and why observations with fMRI scans suggest that brain regions important for attention or executive control are affected after an acute bout of exercise (Weng et al., 2017).

It is difficult to clearly separate the mechanisms of signaling pathways (e.g., BDNF, serotonin) from those of cerebral shifting effects (transient hypofrontality) during physical activity because both occur simultaneously during exercise. Therefore, it is also difficult to attribute the amount of the effect to either the hypofrontality hypothesis or the released factors. One approach has been taken by Budnik-Przybylska et al. (2021) who presented evidence for a transient hypofrontality during self-produced motor imagery (MI). MI is characterized as an internal simulation of a movement without corresponding motor output (Jeannerod, 1994), i.e., without muscle contractions. MI has various effects such as promoting motor learning (Di Rienzo et al., 2016) or leading to strength gains (Yue and Cole, 1992). There are several theories addressing the underlying mechanisms of MI. One is simulation theory in which it is assumed that MI is based on the same representations as those that are also used for motor execution. This theory is referred to in studies examining activation patterns between MI and actual movement in cortical areas (Lotze et al., 1999; Jeannerod, 2001; Munzert et al., 2009). For instance, the frontal motor area has been shown to represent content and modality of both imagined and executed actions (Pilgramm et al., 2016). Nonetheless, although these patterns overlap, they are not identical (Zabicki et al., 2017). Considering these similarities between MI and executed actions, it can be hypothesized that the effects of the hypofrontality hypothesis may also be observed when applying MI. Therefore, it seems feasible to use an MI condition to address cerebral shifting effects (transient hypofrontality) and a physical exercise (PE) condition to address the release of signaling factors (e.g., BDNF, serotonin) due to muscle contraction along with the respective sensory feedback, although these mechanisms are not measured in this study directly.

There are also some inconsistencies in the results of previous studies. These can be explained partly by different groups (e.g., children or elderly; physically trained or untrained participants) but also by different study designs using various intervention protocols. On the one hand, physical activity varies in terms of, e.g., the type of activity (such as walking or swimming), its duration (short or long), and its intensity (submaximal or maximal). On the other hand, the cognitive tests used also vary (e.g., memory or attention). For this reason, Basso and Suzuki (2017) suggested clear and common standards for future studies analyzing acute exercise effects on cognition or behavior. They proposed that researchers should consider and report the parameters listed in Table 1. These are: (1) duration, (2) intensity, (3) perceived exertion, and (4) exercise index. A meta-analysis (Chang et al., 2012) identified three further potential moderators: (5) type of cognitive performance and (6) participants’ fitness. In addition, effects are influenced by (7) timing of testing (e.g., during exercise, immediately following exercise, or after a delay). Therefore, we added these three further parameters to Table 1.

**Table 1:** Recommended acute exercise study standards and values based on Basso and Suzuki (2017, Points 1–4: Duration, Intensity, Perceived exertion, and Exercise index) and Chang et al. (2012, Points 5–7: Cognitive performance, Participants’ fitness, and Timing of testing) and values used in the current study.

Acute exercise measurement and category	Recommended values	Values in current study
(1) Duration (measured in min)		
Short	0–15 min	
Moderate	16–45 min	25 min
Long	46 min or longer	
(2) Intensity (measured in percentage of VO2 max or percentage HR max)	(% VO2 max)	(% HR max)
Low	≤39	
Moderate	40–59	70
High	≥60	
(3) Perceived exertion measured by the Borg Ratings of Perceived Exertion Scale	6–20	6–20
(4) Exercise index a combined value of duration, intensity and perceived exertion	Calculation: (% of hour + % of VO2 max + % of scale)/3	Calculation: (% of hour + % of HR max + % of scale)/3
(5) Cognitive performance	Different cognitive tests	d2-R (sustained attention)
(6) Participants’ fitness	e.g., maximal VO2, daily activity	Activity over last 4 weeks
(7) Timing of testing	During	
	Immediately after	
	With a delay	20-min delay

Based on the above-mentioned literature and the recommendations regarding the study design (Chang et al., 2012; Basso and Suzuki, 2017), the present study aimed to examine the effects of a single bout of PE and of a matched MI endurance session on sustained attention. The idea was to gain further insights into the mechanisms of cognitive functioning after bodily or mental activity. We hypothesized that both interventions—PE and MI—would have positive effects on cognitive performance, with higher increases in sustained attention after PE compared to MI sessions due to the global release of signal factors (such as an increase in BDNF) that can be expected based on the literature. Hence, MI should reflect the improvements due to transient hypofrontality effects, whereas PE should add differential signal factors to the transient hypofrontality effects.

Because previously reported studies chose different designs and did not report all parameters, comparability is difficult. Therefore, by specifying the precise load as well as the test execution parameters in line with the recommendations of Basso and Suzuki (2017) and Chang et al. (2012), this study aims to contribute to a better comparability and interpretation of acute cognitive effects after physical activity. According to the potential moderators (intensity and duration of exercise, as well as cognitive test and timing) for improvement after physical activity identified by Chang et al. (2012) in their comprehensive meta-analysis, the present study is designed to produce potentially high effects.

## Materials and methods

### Participants

Participants were 56 sport students at the University of Frankfurt, Germany. Their age range was 20–35 years (*M* = 23.04 years, SD = 3.14) and 29 were male. The study fulfilled the requirements of the local ethics committee, and informed consent was obtained from all participants prior to any data collection.

### Procedure

After arriving and being informed about the testing protocol, participants gave written informed consent and filled in a German-language physical activity, exercise, and sport questionnaire (BSA 2.0-Fragebogen from Fuchs et al., 2015) to assess their physical activity over the last 4 weeks. Then, all participants performed the d2-R test in a group setting to measure sustained attention performance. To match the experimental groups for their initial level, participants were pairwise matched according to gender and performance in the pre-test.

**Figure 1:**
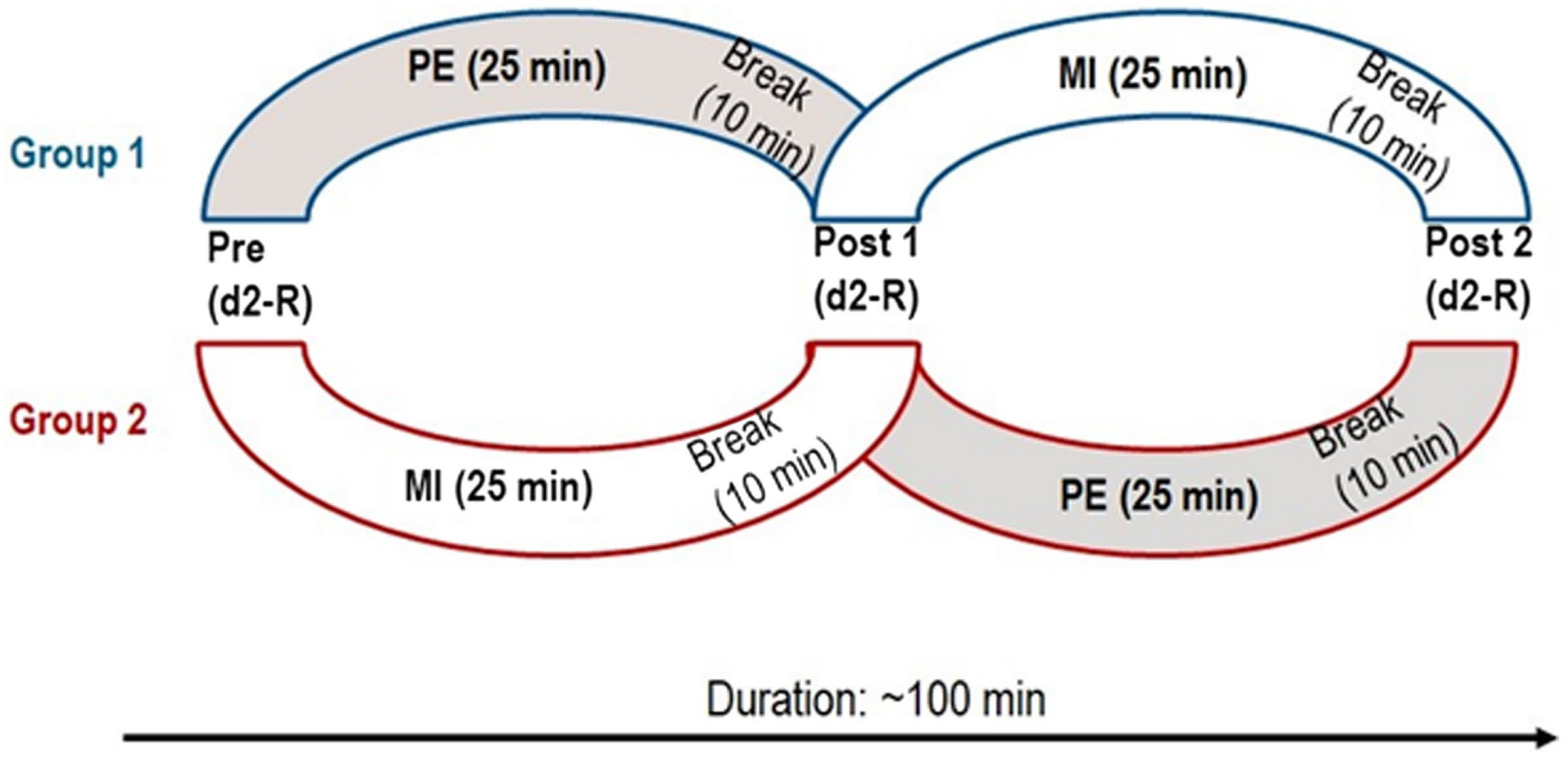
Timeline of the experiment. Group 1 first completed the physical exercise (PE) and then the mental imagery exercise (MI). Group 2 first completed MI and then PE. Both groups completed a test for sustained attention (d2-R) three times.

Both groups completed two moderate training sessions in a crossover design (see Figure 1). One training session consisted of a moderate PE; the other, of a moderate mental exercise (MI) matched for duration. Moderate exercise was chosen to keep the physiological load between PE and MI more comparable than MI to intense exercise. Both sessions lasted exactly 25 min. Precisely 10 min after each moderate training session (PE or MI), participants performed the d2-R test again (Post 1, Post 2). Participants performed the posttests in the same group setting and sitting in the same seats. The d2-R instructions were repeated in exactly the same way before both posttests.

### Physical exercise

The physical endurance exercise was performed on a 400-m track. Each participant’s pace was based on their individual heart rate. As recommended by Basso and Suzuki (2017), the individual maximal heart rate (HR_max_) of each participant was calculated in line with Tanaka et al. (2001) as 208 – (0.7 * age). Heart rate was controlled *via* monitors (Polar F6; Polar Electro, Kempele, Finland). Participants had to adjust their running speed to run at a moderate intensity of 70% of individual HR_max_. All participants were familiar with pacing their running speed with the help of heart rate monitoring from endurance classes. A research assistant recorded and verified time and running speed after each lap (400 m) according to their individual HR and gave feedback about their pace. They also recorded the total running distance at the end of the session. Total running distance was used as an indicator of physical performance: the longer the running distance, the better the endurance performance. As a manipulation check, we asked students to report their rate of perceived exertion (RPE) on the Borg scale from 6 to 20 (corresponding to a normal heart rate range of 60–200) used to measure overall exertion during physical activity (Borg and Kaijser, 2006). We used the data from the Borg scale, the duration of exercise sessions, and heart rate to calculate an exercise index based on Basso and Suzuki’s (2017) equation.


$$\mathrm{Exercise index}=\left(\begin{array}{l} \%\;\mathrm{of hour for session}\\ +\;\%\;\mathrm{of}\;{HR}_{\text{max}}+\%\;\mathrm{of scale} \end{array}\right)/3$$

### Motor imagery

The MI session was developed from Holmes and Collins’s (2001) PETTLEP model (Physical, Environment, Task, Timing, Learning, Emotion, Perspective). The instructions describe the process of a moderate mental running session from a first-person perspective. Participants were located on the track and each received their medium-intensity instructions *via* a sound recording. During the 25-min MI session, students sitting on the track felt the track under their feet, but were not allowed to move or run. To check their imagery, 5 min after the MI session, participants were asked to describe the amount of time they spent focused on the imagery task (in percent) and to rate the vividness of their overall motor imagery session (Zabicki et al., 2017) on a Likert scale from 1 (*absolutely clear and lively*) to 5 (*absolutely no imagination*) in the format of the VMIQ-2 (Roberts et al., 2008).

### Sustained attention test

We measured sustained attention with the d2-R test (Brickenkamp et al., 2010), a paper-and-pencil test in which participants mark special letters under time pressure. In 14 lines with 47 letters, the letters “p” or “d” are presented in a randomized order with one, two, three, or four vertical stripes either above or below each “p” or “d.” The instructions are to mark the letter “d” with two stripes, to ignore those with one, three or four stripes, and work as quickly and correctly as possible. Each line has a 20-s time limit, and because there is no break between lines, the total test time is 4 min and 40 s. We administered the d2-R as a group test. The dependent variable was sustained attention performance, which is composed of the marked targets (letter “d” with two strikes) and errors (omitting a “d” with two strikes or marking a false one). The final score is calculated according to an age reference with a maximum score of 130. Participants performed the test in a lecture hall with at least two or more vacant seats to their left and right.

### Statistical analyses

Statistical analyses were performed with the software SPSS (IBM SPSS Statistics for Windows Version 25) and alpha was set at 0.05 for all statistical tests. Data were tested for normal distribution and homogeneity of variance using Shapiro–Wilk and Levene tests prior to the statistical analyses. To control for carryover effects, a specifically recommended unpaired *t*-test as suggested by Wellek and Blettner (2012) was calculated. We applied a Greenhouse–Geisser adjustment for violation of sphericity. Two repeated-measures analyses of variance (ANOVAs) were computed to examine the effects of condition (MI, PE) over time (pre, post) and the effects of group (MI-first, PE-first) over time (Pre, Post 1, Post 2). For post hoc comparison, the percentage change score between Pre to Post 1 and Post 1 to Post 2 was calculated for both groups and compared using an independent *t*-test. The effect size of the respective *t*-tests was reported with Cohen’s d. Additionally, the effect size of the PE compared to the MI intervention from Pre to Post 1 was calculated using the mean values and standard deviation of Pre and Post 1 and the number of participants of each group with the freeware of Lenhard and Lenhard (2016).

## Results

Three participants were excluded from the analyses: two due to wrong test processing of the d2-R, and one due to technical difficulties with the heart-rate monitor. The 53 analyzed persons were divided into Group 1 (that started with PE) with 26 persons (*M* = 23.00 years, SD = 3.73, 14 male) and Group 2 (that started with MI) with 27 persons (*M* = 23.10 years, SD = 2.21, 15 male). Participants reported a mean of 1,523.9 (SD: ±856.5) min of sporting activity during the last 4 weeks and ran an average of 3,392.5 (SD: ±443.3) meters during the 25-min PE session. Based on the World Health Organization (2022) recommendations of 75–150 min of physical activity per week (results in 300–600 min over 4 weeks) for adults aged 18–64 years, the participants in this study are physical active for more than twice the time and therefore can be classified as physical active. RPE for the PE session was rated at a mean of 9.6 (SD: ±1.9) and the average heart rate was 142.9 (SD: ±10.9; target value: 143.9 ± 1.7) bpm. The calculated exercise index was an average of 45.89 (SD: ±4.74). MI sessions were rated as being moderately clear and vivid (*M* = 3.2 points from a maximum of 5 points, SD: ±0.9), and participants reported that they focused *M* = 55.1% (SD: ±22.2) of the time on imagery.

### Comparison between conditions

**Figure 2:**
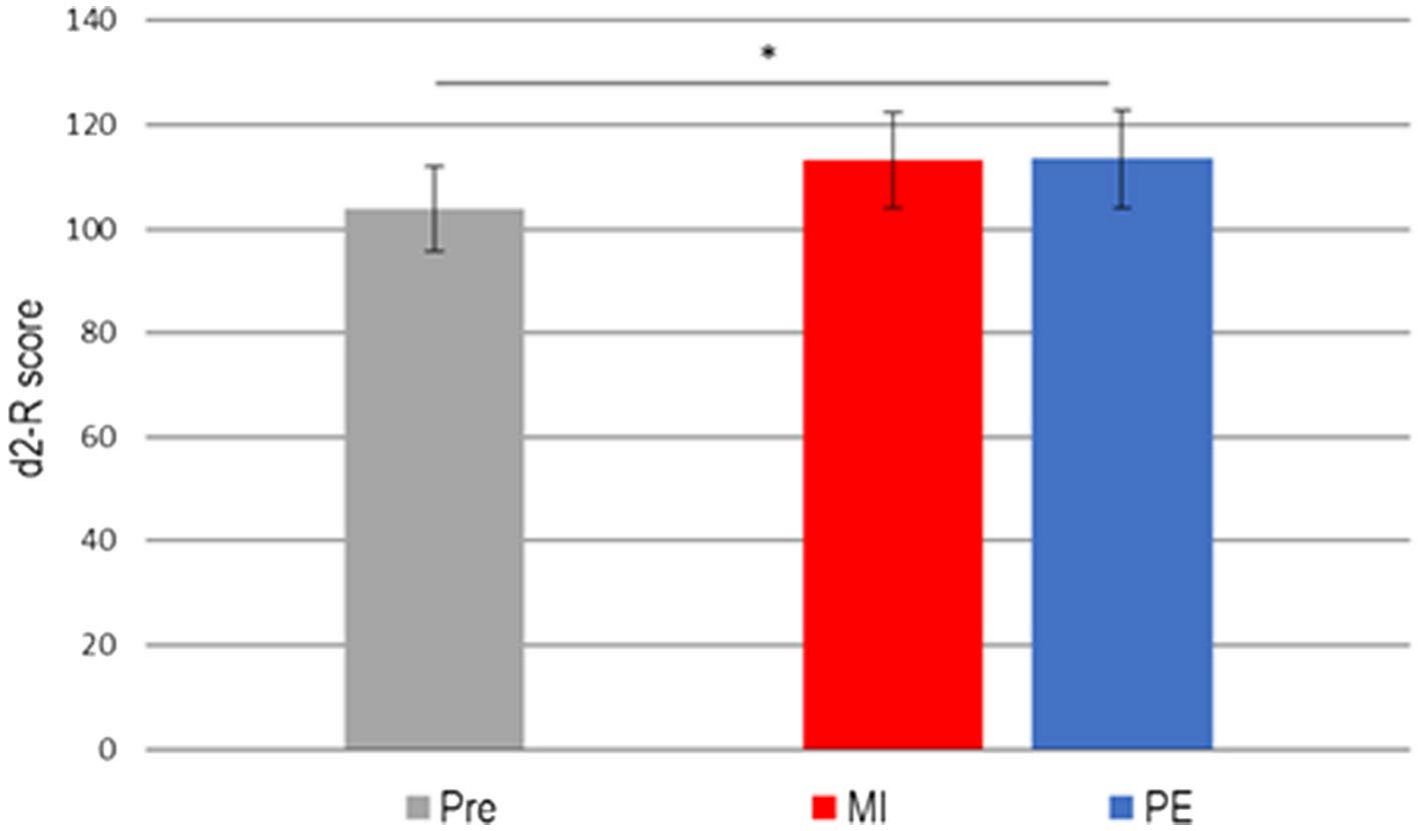
Mean (±SD) d2-R score before (Pre) and after MI or PE exercise. Significant difference (^*^p < 0.05) between pre and both post MI and post PE.

Test for carryover effects showed no significant differences, *t*(51) = −1.29, *p* = 0.20, *d* = 0.35, which is relevant for crossover designs to yield valid results. The 2 × 2 (Condition × Time) ANOVA showed no interaction effect*, F*(1, 52) = 0.05, *p* = 0.82, *η*^2^ = 0.001, and no significant main effect for condition, *F*(1, 52) = 0.05, *p* = 0.82, *η*^2^ = 0.001, but a significant main effect of time, *F*(1, 52) = 265.89, *p* < 0.001, *η*^2^ = 0.84 (see Figure 2).

### Comparison between groups

**Figure 3:**
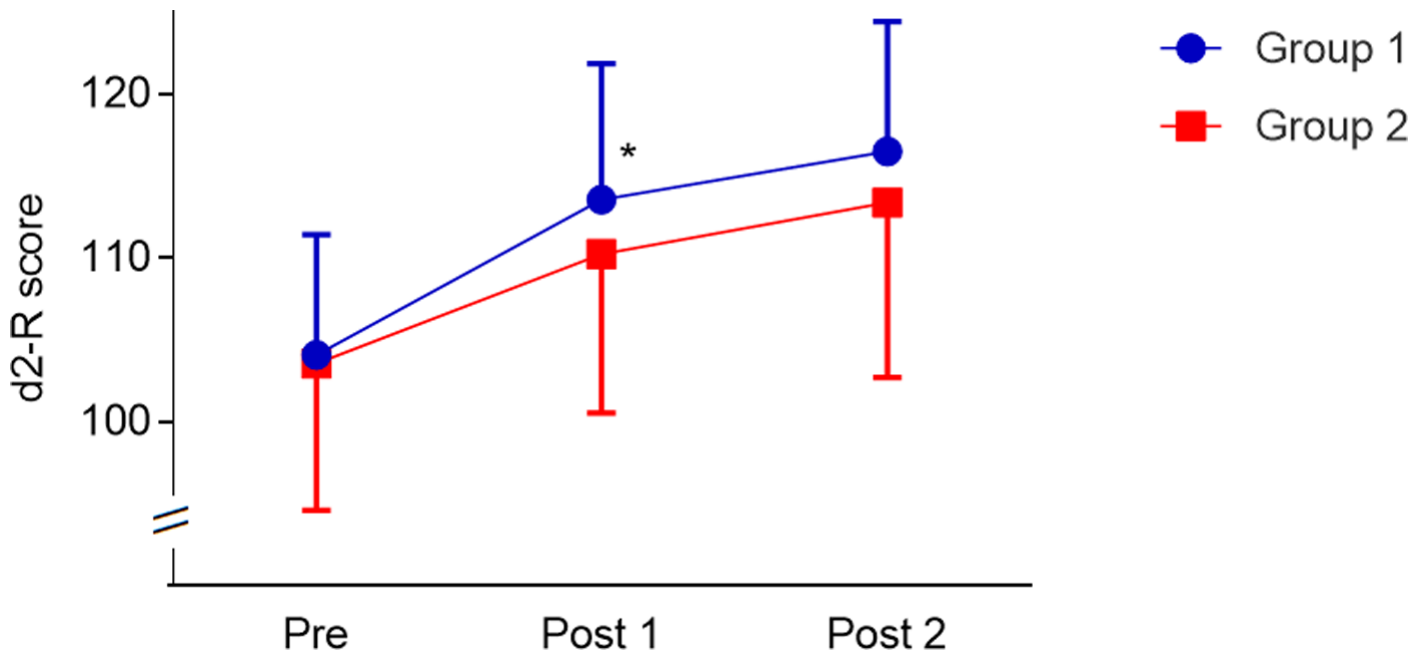
Change in d2-R score (Mean ± SD) of both groups (Group 1, PE First; Group 2, MI first) over time. Significant interaction (^*^p < 0.05) between time and group with higher score for Group 1 at Post 1.

Mauchly’s sphericity test indicated that the assumption of sphericity had been violated for the score in d2-R over time (Pre, Post 1, Post 2), *χ*^2^(2) = 14.01, *p* = 0.001. The 2 × 3 (Group × Time) ANOVA with a Greenhouse–Geisser correction revealed a significant interaction, *F*(1.6, 81.9) = 3.64, *p* = 0.04, *η*^2^ = 0.07 and a significant main effect time, *F*(1.6, 81.9) = 197.03, *p* < 0.01, *η*^2^ = 0.79, but no main effect group, *F*(1, 51) = 0.97, *p* = 0.33, *η*^2^ = 0.02. Prescores of both groups were not different (*t*(51) = −0.23, *p* = 0.82, *d* = 0.07), but improvement from Pre to Post 1 was higher for the PE-first group (9.1%) than for the MI-first group (6.4%; *t*(51) = −2.71, *p* = 0.09, *d* = 0.75). Further increases from Post 1 to Post 2 did not differ between both groups (*t*(51) = 0.19, *p* = 0.85, *d* = 0.05): 2.9% (PE-first) and 2.6% (MI-first, see Figure 3).

## Discussion

The present study examined the effects of a single session of physical endurance exercise (PE) as well as the effects of a single session of an imagined endurance exercise (MI) on a sustained attention task with the aim of gaining further insights into the mechanisms underlying exercise-induced cognitive improvements. In this context, the MI condition reflects improvements based on cerebral shifting effects, whereas the PE condition reflects improvements based on various mechanisms that include cerebral shifting, but in addition others such as global or local release of signaling factors that act on pathways for functional and structural cerebral changes.

We hypothesized improvements in both conditions, but greater improvements after the PE intervention because this involved both mechanisms. At first glance, the primary finding of the study is an enhanced cognitive performance in the d2-R for both conditions (PE & MI), but due to the known retest effects in cognitive tests (d2-R; Schmidt-Atzert et al., 2021), results must be examined in more detail. Because we investigated retest effects with two instead of three measurement points, results were analyzed within groups (i.e., in the order Pre, Post 1, and Post 2 with the different starting conditions PE-first vs. MI-first) instead of using averaged MI and PE values for both measurement times. In addition to the main effect of time, this analysis reveals an interaction effect between time and group. Prescores of both groups did not differ, but improvement from Pre to Post 1 is higher (9.1%) in the PE-first group than in the MI-first group (6.4%). Further increases from Post 1 to Post 2 are lower and similar in size for both groups: 2.9% (PE-first) and 2.6% (MI-first). Comparing improvements in the MI-first group after the first intervention (i.e., after MI) with values addressing retest effects under comparable conditions (similar baseline score and time between both test executions; see Brickenkamp et al., 2010) reveals a percentage improvement of 5.9%. This comparison suggests that the MI intervention does not show any additional effect going beyond the retest effects, and that only those with PE in the first session benefit cognitively from their activity. After the second session, both groups show a low improvement from Post 1 to Post 2 (2.9% and 2.6%), but the groups do not differ. We hypothesize that the absence of an effect due to PE could be explained by a ceiling effect of repeated testing. Which means, according to the compensation theory (Karbach and Unger, 2014) that individuals benefit fewer from cognitive training if the room for improvement is less. As participant’s starts with a higher score into the second intervention phase, the level for improvement is less for both groups, which could be why PE cannot achieve the expected results for the MI first group during the second intervention phase. Unfortunately, we cannot draw on results from other studies investigating d2-R to confirm this hypothesis. Another explanation for missing effects that cannot be excluded are cognitive fatigue effects. However, due to the short test duration of the d2-R (4:40 min and in total 14 min), we rather assume low fatigue effects. Furthermore, a carryover effect of the physical activity on the second mental activity cannot be completely excluded. Although the statistical evaluation as well as the expected rapid decrease in heart rate (Pierpont et al., 2000) and heart rate variability (Scott et al., 2017) after moderate physical activity speaks against a washout phase that is too short, signaling factors or other mechanism may still be slightly active. This resulted in the lack of a second improvement after PE, which meant that the analysis of the conditions (PE and MI) did not reveal any significant differences. Therefore, we assume that only PE has a positive effect on sustained attention, and that MI does not. This would suggest that cognitive improvement is more likely to be enhanced by PE, which have been hypothesized to induce a variety of neurobiological mechanisms and not, or at least not only, by a redistribution of neuronal activity in the brain. Possible mechanisms or signaling pathways after PE could be a global release of signaling factors (e.g., neurotransmitter or growth factors), a mediation of brain mechanism, like the orexin system (Chieffi et al., 2017) or an increased blood flow to the brain, which increases the oxygen uptake (Ogoh and Ainslie, 2009).

However, we still need to mention aspects that could explain the absence of effects due to MI, which should induce transient hypofrontality effects. For example, the intensity could have been slightly too low. Indeed, participants show higher performance on prefrontal-cortex-dependent tasks after high-intensity rather than medium-or low-intensity exercise (Crawford and Loprinzi, 2019; Loprinzi et al., 2019). Furthermore, the delay between exercise and cognitive test in our study may be too long (10 min between ending the activity and starting the test) for transient hypofrontality effects, because most studies addressing transient hypofrontality theory tested during exercise or with only a short delay (for a review, see, e.g., Jung et al., 2021). Another aspect is that high physical fitness is a potential moderator for prefrontal-cortex-dependent tasks, and that effects will be lower for highly trained participants (Jung et al., 2021). This could impact on our group of physically rather active participants. However, there is also evidence that participants’ individual fitness status is related to an increase in BDNF level after exercise, meaning there is a greater increase in BDNF in trained compared to untrained subjects (Tsai et al., 2016). A higher BDNF level after acute exercise is related, in turn, to better cognitive performance (Winter et al., 2007). This suggests an advantage for the PE condition, since our group includes physically active people. In this context, BDNF represents the signaling factor leading to enhanced cognitive performance after acute physical activity. As mentioned in the introduction, there are other potential factors such as serotonin or IGF-1, but their differentiation was not the goal of our study. We aimed to compare possible cerebral shifting effects against the overall effects after moderate physical activity. Taking into account the above-mentioned constraints, our results indicate that the global release of neurotransmitter or growth factors is necessary for cognitive improvements with a delay after physical exercise.

The effects of physical exercise on sustained attention can be shown in the results from Pre to Post 1. The effect size of PE (MI as control group, calculated with absolute values) from Pre to Post 1 is *d* = 0.34 indicating a small positive effect of physical exercise on sustained attention (Lenhard and Lenhard, 2016). This is in line with the outcomes of meta-analyses that also report a small positive effect in studies measuring cognition after exercise (Lambourne and Tomporowski, 2010; Chang et al., 2012). Looking at effect sizes more closely, it can be seen that our study reveals a slightly larger effect than that in Chang et al.’s (2012) and Lambourne and Tomporowski’s (2010) meta-analysis (*g* = 0.10, *d* = 0.20). This might be due to the design of the study and the aforementioned potential moderators, which were based on the results of Chang et al.’s (2012) meta-analysis and Basso and Suzuki’s (2017) recommendations (see Table 1), leading to the detection of larger effects. Chang et al. (2012) reported that the duration, the intensity of the load, and the timing of the test influence effects. They stated that at least 20 min of exercise are necessary to see these effects, but that protocols lasting much longer than 20 min are subject to side effects such as fatigue or dehydration. This is why we chose 25 min for our intervention time. Another important issue seems to be the timing of test administration for cognitive function, with largest effects found with a delay of about 11–20 min and a flattening of the effects after 20 min. For intensity, there is some evidence that higher intensities lead to greater effects with a delay after exercise. For lower intensities, the effect is measurable for only a very short time or immediately after the end of the activity. Because we chose a medium intensity in order to ensure every participant would be able to complete the 25 min of endurance activity and keep the physiological load of PE better comparable to MI than with intense exercise, we decided to use a delay time of 10 min. In contrast to Basso and Suzuki (2017), we did not control the intensity *via* percentage of VO2_max_, but *via* percentage of HR_max_ to make our study easier to implement. This is also a valid method for submaximal intensity (Ainsworth et al., 2015). We report perceived exertion as recommended: Values on the Borg scale show that perceived exertion is very light to light. Considering the selected intensity of 70% of HR_max_, however, we would have expected higher values. The low values may be explained by our participants’ relatively high level of fitness. A high fitness level, in turn, acts as a moderator for higher effects on cognitive functions after an acute bout of exercise (Chang et al., 2012). Therefore, we can expect a higher effect in our trained participants. In Chang et al.’s (2012) meta-analysis, greater improvements were measured in attention tests, which is why we chose a test for sustained attention in the current study. To improve comparability between intervention studies examining acute effects on cognitive functions after a single bout of exercise, we calculated and reported an exercise index. The clear presentation and consideration of the load parameters and possible moderators can be used for further analyses or follow-up studies, and results in a comparatively high effect. As more studies report the exact parameters of the intervention and calculate the exercise index according to Basso and Suzuki (2017), the linkage between load and effect can be drawn better.

Finding a good linkage between load parameters and cognitive improvements on acute measurements may also provide conclusions for long-term improvements, as these may result from accumulated acute effects. Indeed, two studies looking for long-term improvements in sustained attention with the same cognitive test as in this study showed higher effect sizes with *d* = 0.70 (Stroth et al., 2009) and *d* = 0.71 (Lehmann et al., 2020) compared to the shown acute effects in this study.

Overall, we conclude that MI, which is supposed to be associated with the transient hypofrontality hypothesis, has no effect on sustained attention when tested with a 10 min delay. PE, which is supposed to be associated with several mechanisms such as cerebral shifting or the global release of signaling factors, etc., leads to an increase in sustained attention—at least after one test, but not on repeated tests. The calculated effect size is in line with the literature. In order to prevent ceiling effects, future studies should choose a test with lower retest effects or not use a crossover design. By describing the load and cognitive test parameters in detail and implementing two different interventions, this study is relevant for future studies or meta-analyses aiming to gain further evidence on the mechanistic effects of acute exercise on cognition.

## Conclusion

The aim of the study was to examine cognitive functioning after a physical and a mental endurance exercise session. Results show improvements for both groups over time, whereby the improvements in the mental group are due to retest effects. However, a greater increase in cognitive functioning after physical endurance exercise emerges when it is operationalized as the first session, suggesting that release of signaling factors such as neurotransmitter or growth factors due to muscle contraction and real sensory feedback processing are further mediating mechanisms going beyond transient hypofrontality that improve cognitive performance.

## Data availability statement

The raw data supporting the conclusions of this article will be made available by the authors, without undue reservation.

## Ethics statement

The studies involving human participants were reviewed and approved by Goethe University Frankfurt am Main, Department of Psychology and Sports Science, Ethics committee. The patients/participants provided their written informed consent to participate in this study.

## Author contributions

BW, M-TF, and KZ designed the experiment, analyzed and interpreted the data, and prepared the manuscript. BW and M-TF performed the experiment. KZ additionally edited the manuscript with the help of a native speaker. All authors contributed to the article and approved the submitted version.

## Conflict of interest

The authors declare that the research was conducted in the absence of any commercial or financial relationships that could be construed as a potential conflict of interest.

## Publisher’s note

All claims expressed in this article are solely those of the authors and do not necessarily represent those of their affiliated organizations, or those of the publisher, the editors and the reviewers. Any product that may be evaluated in this article, or claim that may be made by its manufacturer, is not guaranteed or endorsed by the publisher.
